# Ophthalmia neonatorum due to *Escherichia coli*: A rare cause or an emerging bacterial etiology of neonatal conjunctivitis?

**DOI:** 10.1002/ccr3.6201

**Published:** 2022-08-05

**Authors:** Stephanie Saadeh‐Jackson, Linnet Rodriguez, Christopher T. Leffler, Casey Freymiller, Elizabeth Wolf, Niran Wijesooriya, Natario L. Couser

**Affiliations:** ^1^ Virginia Commonwealth University School of Medicine Richmond Virginia USA; ^2^ Department of Ophthalmology Virginia Commonwealth University School of Medicine Richmond Virginia USA; ^3^ Department of Pediatrics Virginia Commonwealth University School of Medicine, Children's Hospital of Richmond at VCU Richmond Virginia USA; ^4^ Department of Human and Molecular Genetics Virginia Commonwealth University School of Medicine Richmond Virginia USA

**Keywords:** bacterial conjunctivitis, conjunctivitis due to *Escherichia coli*, neonatal conjunctivitis, neonatal prophylaxis, ophthalmia neonatorum

## Abstract

Since the introduction of universal gonococcal and chlamydia prophylaxis, other etiologies for neonatal conjunctivitis such as *Escherichia coli* have become more common. Early eye culturing as part of the management plan could provide swifter treatment and preservation of vision potential in affected neonates.

## Graphical abstract

It is important to develop a diagnostic decision tree for ophthalmia neonatorum secondary to nontraditional bacterial causes such as *Escherichia coli* and to confidently identify their associated clinical presentations in order to ensure swift treatment and preservation of vision potential among neonates.

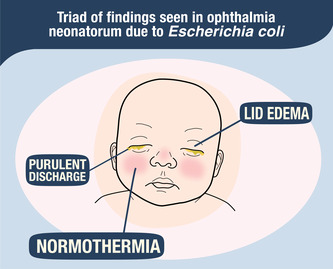

## 1 INTRODUCTION

*Ophthalmia neonatorum*, or neonatal conjunctivitis (NC), is the most common disease of the eye for newborns during the first 4 weeks of life and typically results from exposure to bacteria during birth.^1^ The introduction of topical prophylaxis in neonates has decreased the incidence of N. gonorrhea considerably to <1% in developed countries.^2^ Other bacteria, including *Staphylococcus* species, *Streptococcus* species, *Haemophilus* species, and other Gram‐negative bacteria have become more common, constituting 30%–50% of cases reported in the United States.^3^, ^4^ The U.S. Preventative Service Task Force and American Academy of Ophthalmology have outlined treatment guidelines for *Neisseria gonorrhoeae* and *Chlamydia trachomatis*.^5^ However, with the changing prevalence of various bacterial etiologies, The American Academy of Pediatrics has noted that there is a need for revision of guidelines on the management of neonatal conjunctivitis.^6^

In this study, we present a 7‐week‐old male newborn with neonatal conjunctivitis due to *Escherichia coli*, and we detail the management and treatment approach taken by the medical and ophthalmology team. Additionally, we compare his clinical presentation to the few previously reported cases in the literature and discuss this infectious etiology as a potential emerging cause of neonatal conjunctivitis.

## 2 CASE REPORT

Conceived through in‐vitro fertilization, our patient was born prematurely at 36 weeks, 1 day as the first twin via a vaginal delivery, with APGAR scores of 9 and 9 at 1 and 5 minutes of life, to a Gravid 2 Para 3 39‐year‐old mother with normal prenatal labs including negative Group B streptococcal screening. Her gonorrhea and chlamydia history was unknown as there was no documented prenatal care records in her chart. The mother reported no sexually transmitted infections or diseases during the course of the pregnancy, and both mother and father were otherwise reported to be healthy and without any particular medical conditions. Family history was unremarkable. The pregnancy was complicated by induction of labor due to decreased fetal movement, cholestasis, and preeclampsia without severe features. At birth, he weighed 2.75 kg, measured 49 cm in length, and had a head circumference of 34 cm, all of which were appropriate for his gestational age. The newborn exam was significant only for bilateral calcaneovalgus deformity. Routine newborn care through vitamin K administration and erythromycin 0.5% ointment application in both eyes was followed per standard of care. He had a brief and isolated episode of hypothermia upon admission into the nursery unit, with no other concerning vital signs or clinical symptoms on presentation, which was soon resolved when the patient was placed under a radiant warmer. To ensure the isolated episode of hypothermia was only due to environmental factors, a complete blood count (CBC) and blood culture were obtained on the second day of life to rule out sepsis. After adjusting for the newborn's age, the CBC was normal, and the blood culture later showed no growth. Given our patient's reassuring immature neutrophil‐to‐total neutrophil ratio as derived from the CBC (0.15), sepsis was ruled out, and broad‐spectrum antibiotics were not pursued.

On the second day of life, the patient developed copious opaque yellow discharge in the right eye with crusting of eyelashes, and the pediatric ophthalmology service was consulted. The ophthalmic exam revealed mild injection of the palpebral conjunctiva of the right eye, along with mild edema, erythema, and yellow discharge of the right lids. No other significant exam findings were noted while evaluating the optic nerve and fundus of both eyes, and there were no notable exam findings for the left eye. With *ophthalmia neonatorum* suspected, a gram stain and culture were obtained from the discharge of the right eye. In the meantime, it was recommended to rinse the right eye four times daily with a saline lavage, along with continuation of the erythromycin ointment four times daily for the right eye and the administration of ceftriaxone 70 mg IM once daily for suspected gonococcal conjunctivitis for 2 days. A prescription of oral erythromycin 50 mg/kg/day in four divided doses for 14 days was also initiated and administered every 6 h.

On the third day of life, the gram stain of the eye discharge revealed Gram‐negative rods, so oral erythromycin was discontinued after the primary team determined from this that *Chlamydia trachomatis* was not the causative organism. A second dose of IM ceftriaxone was given prior to discharge from the newborn nursery unit, and the erythromycin ointment was continued after discharge to maintain treatment for *Neisseria gonorrhoeae* until the patient's outpatient follow‐up appointment with ophthalmology.

The following day, the cultures revealed *Escherichia coli* susceptible to ampicillin, cefazolin, cefepime, ceftriaxone, ciprofloxacin, gentamicin, levofloxacin, meropenem, ofloxacin, piperacillin/tazobactam, and trimethoprim/sulfamethoxazole. The organism was resistant to bacitracin. The family was contacted and instructed to stop the erythromycin ointment and start using levofloxacin ophthalmic solution, one drop four times daily to the right eye.

On the sixth day of life, the patient presented to outpatient ophthalmology for follow‐up and was noted to have improved but persistent right eye crusting, along with new‐onset left eye crusting that prompted the parents to start levofloxacin drops in that eye as well. The chlamydial culture returned 7 days after birth and was negative. At this time, it was recommended to continue the levofloxacin drops with one drop in both eyes four times daily for 7 days, and the patient was to return to outpatient ophthalmology in 1 week for follow‐up.

On the 12th day of life, the patient's ophthalmic symptoms and signs had resolved.

## 3 DISCUSSION

Based on previous case reports and the clinical findings in our case (see Tables 1 and 2), we conclude that features of ophthalmia neonatorum due to *E. coli* include the following: onset within the first 3 days of life, normothermia, purulent discharge, and lid edema. The role of maternal genitourinary infections is unclear. One study found a statistically significant association between *E. coli* conjunctivitis and a higher rate of positive history of genitourinary infections in their mothers,^7^ although this was not the case with our patient. This same study also found a statistically significant association between neonatal septicemia and neonates with *E. coli* conjunctivitis.^7^ Although neonatal conjunctivitis is widely considered to be due to *Neisseria gonorrhoeae* or *Chlamydia trachomatis*, assembling the clinical picture and associations for *E. coli* conjunctivitis will allow swifter treatment of the ailing newborn's eyes and prevent long‐term damage to their vision, especially those of newborns experiencing sepsis. It is important to additionally recognize that, within the neonatal age group, any focal infection can prompt concern for sepsis. Thus, if a neonate presents with conjunctivitis as our patient did, pursuing sepsis work‐up is appropriate in order to rule out the need for empiric antibiotic treatment for sepsis.

**TABLE 1:** Clinical presentation of ophthalmia neonatorum due to *Escherichia coli* in three newborns

Author of and Year Article Published	Gestational age	M/F	Method of Delivery	Time to Onset Since Birth	Affected Eye(s)	Discharge	Time to Complete Resolution	Treatment
Current case report	36.1 wks	M	Spontaneous vaginal delivery	1 day	Unilateral→Bilateral	Yellow, opaque, copious, purulent	1 week	Levofloxacin 0.5% drops with 1 drop every 4 h in both eyes daily, not to exceed 4 doses per day, for 7 days
Maalouli and Pitt 2017^9^	39 wks	F	Spontaneous vaginal delivery	3 days	Unilateral	Purulent	12 days	Moxifloxacin 0.5% 1 drop to each eye 3 times daily for 7 days
Golshani et al 2013^10^	“Full term”	M	Spontaneous vaginal delivery	17 hours	Bilateral	Purulent	Unspecified	Moxifloxacin 0.5% ophthalmic drops four times a day in both eyes (Unspecified medication strength and duration)

**TABLE 2:** Additional clinical findings associated with ophthalmia neonatorum due to *Escherichia coli* in three newborns

	First 24 hours of life	Normothermia	Purulent Discharge	Lid Edema	GC/Chlamydia
Current case report	−	+	+	+	Unknown/Unknown
Maalouli and Pitt 2017	−	+	+	+	Unknown/Unknown
Golshani et al 2013	+	+	+	+	−/−

Given that maternal genitourinary infection during pregnancy seems to be a potential contributor to neonatal conjunctivitis due to *E. coli,* future directions within this area of research could involve retrospective chart review of all mothers who have delivered newborns with such conjunctivitis and subsequent identification of the most common infectious etiologies for identification of trends in bacterial etiology of neonatal conjunctivitis.

Finally, this case report reflects the importance of adapting management decisions to unique etiologies of a common disease. Saline irrigation is commonly used to remove the debris that accumulates in response to infection.^8^ In addition to this, if the patient does not have symptoms that align with the clinical presentations for either *Chlamydia trachomatis* or *Neisseria gonorrhoeae,* the primary team should obtain eye cultures prior to initiating empiric treatment for the suspected pathogen. Introducing topical quinolones while awaiting culture results to prophylactically treat for *E. coli* could then prevent any impact on the newborn's vision potential.

## AUTHOR CONTRIBUTIONS

Stephanie Saadeh‐ Jackson, Linnet Rodriguez, Christopher Leffler, Casey Freymiller, Elizabeth Wolf, Niran Wijesooriya, Natario Couser: Made substantial contributions to conception and design, or acquisition of data, or analysis and interpretation of data, was involved in drafting the manuscript or revising it critically for important intellectual content, gave final approval of the version to be published, and agreed to be accountable for all aspects of the work in ensuring that questions related to the accuracy or integrity of any part of the work are appropriately investigated and resolved.

## CONFLICT OF INTEREST

Stephanie Saadeh‐Jackson, MD, MS: None.Linnet Rodriguez, MD: None. Christopher T. Leffler, MD MPH: None. Casey Freymiller, MD: None. Elizabeth Wolf, MD, MPH: None. Niran Wijesooriya, MD: None. Natario L. Couser, MD, MS: (1) Retrophin, Inc./Travere Therapeutics, Inc. (Clinical Trial). (2) National Cancer Institute/Children's Oncology Group (Clinical Trial). (3) Elsevier (Book editor). (4) Patient‐Centered Outcomes Research Institute (PCORI; Advisory Panel on Rare Disease).

## ETHICS APPROVAL

The authorship team is in agreement that this manuscript upholds the guidelines outlined by this journal regarding research integrity and publishing ethics.

## CONSENT

Written consent has been obtained.

## Data Availability

None. Data sharing is not applicable to this article as no datasets were generated or analyzed for the current report.
